# Targeted Therapy of Acute Liver Injury via Cryptotanshinone-Loaded Biomimetic Nanoparticles Derived from Mesenchymal Stromal Cells Driven by Homing

**DOI:** 10.3390/pharmaceutics15122764

**Published:** 2023-12-12

**Authors:** Xin Zhang, Yao Yi, Yuanyuan Jiang, Jinqiu Liao, Ruiwu Yang, Xuexue Deng, Li Zhang

**Affiliations:** 1 College of Science, Sichuan Agricultural University, Ya’an 625014, China; 2021115002@stu.sicau.edu.cn (X.Z.); 2021215012@stu.sicau.edu.cn (Y.Y.); 14273@sicau.edu.cn (Y.J.); x.x.deng@sicau.edu.cn (X.D.); 2 College of Life Science, Sichuan Agricultural University, Ya’an 625014, China; liaojinqiu630@sicau.edu.cn (J.L.); yrwu@sicau.edu.cn (R.Y.)

**Keywords:** acute liver injury, mesenchymal stromal cells, biomimetic drug delivery system, cryptotanshinone

## Abstract

Acute liver injury (ALI) has the potential to compromise hepatic function rapidly, with severe cases posing a considerable threat to human health and wellbeing. Conventional treatments, such as the oral administration of antioxidants, can inadvertently lead to liver toxicity and other unwanted side effects. Mesenchymal stromal cells (MSCs) can target therapeutic agents directly to inflammatory sites owing to their homing effect, and they offer a promising avenue for the treatment of ALI. However, the efficacy and feasibility of these live cell products are hampered by challenges associated with delivery pathways and safety concerns. Therefore, in this work, MSC membranes were ingeniously harnessed as protective shells to encapsulate synthesized PLGA nanoparticle cores (PLGA/MSCs). This strategic approach enabled nanoparticles to simulate endogenous substances and yielded a core–shell nano-biomimetic structure. The biomimetic nanocarrier remarkably maintained the homing ability of MSCs to inflammatory sites. In this study, cryptotanshinone (CPT)-loaded PLGA/MSCs (CPT@PLGA/MSC) were prepared. These nanoparticles can be effectively internalized by LO2 cells. They reduced cellular oxidative stress and elevated inflammatory levels. In vivo results suggested that, after intravenous administration, CPT@PLGA/MSCs significantly reduced uptake by the reticuloendothelial system and immune recognition compared to PLGA nanoparticles without MSC membrane coatings, subsequently resulting in their targeted and enhanced accumulation in the liver. The effectiveness of CPT@PLGA/MSCs in alleviating carbon tetrachloride-induced oxidative stress and inflammation in a mouse model was unequivocally demonstrated through comprehensive histological examination and liver function tests. This study introduces a pioneering strategy with substantial potential for ALI treatment.

## 1. Introduction

Acute liver injury (ALI) is a common pathological condition caused by viruses, bacteria, alcohol, and drugs [1,2]. It is characterized by rapid changes in liver cell structure, including fat degeneration, intensified oxidative stress, inflammatory responses, and liver cell apoptosis or necrosis, within a relatively short period of time [3,4,5]. ALI inevitably leads to reductions in overall health and is life-threatening due to its acute and destructive nature [6]. Antioxidant agents, such as *N*-acetylcysteine, have shown preventive and therapeutic effects against ALI. However, their clinical application is considerably limited by their rapid excretion and suboptimal drug accumulation at the target site, resulting in poor bioavailability [7,8]. Although approved drugs on the market, such as silymarin and acetaminophen, demonstrate effectiveness, they may induce liver toxicity and other side effects, including endocrine system disruption and neurological abnormalities [9,10]. Therefore, given the challenges associated with ALI, employing an efficient and low-toxicity liver-targeting drug delivery system may represent an effective approach for its targeted therapy.

Liposomes are the first nano drug delivery systems that were successfully translated into real-time clinical applications [11]. However, traditional nanocarriers for drug delivery suffer from inherent drawbacks, such as poor exogenetic origin and biocompatibility [12,13] and short half-lives [14]. These limitations lead to their easy clearance by macrophages, remarkably restricting their application and development in clinical treatments for ALI. In recent years, research on cell membrane-coated biomimetic nanocarriers has attracted widespread attention and provided novel design strategies for achieving safe and efficient drug delivery and translation in ALI treatment. In contrast to conventional delivery systems, cell membrane-coated biomimetic nanocarriers combine the material compositions of cell membranes with the structural and functional features of multifunctional core nanocarriers. This combination not only enables the biomimicry of biological functions, such as endogenous stealth properties, immune evasion [15], and specific recognition avoidance [16], but it also provides excellent biocompatibility.

Cell membrane-coated biomimetic nanocarriers, a simple top-down innovative technology, utilize cell membranes as carriers to transport functionally diverse core nanomedicine, thus facilitating the active targeting of lesions and site-specific drug release [17]. Among nanocarriers, the MSC membrane drug delivery system has garnered increasing attention from researchers. This system involves modifying drug surfaces with MSC membranes and conferring drug carriers with the various biological activities of MSCs, allowing for enhanced flexibility in carrier application and improved circulation stability, thus resulting in precise drug delivery to the target site. MSC delivery systems are distinguished from other drug delivery systems, such as passive targeting by liposomes, by the homing properties of MSCs that allow for the specific recognition of target cells through membrane surface signaling molecules [18]. Chemokines and cytokines activate various pathways to stimulate stem cell homing, and thus, MSCs actively target the desired tissues and accurately regulate the functional state of the target cells [19]. CD44 plays a key role in the homing effect of MSCs [20], and research has shown that stimulating its expression by using hyaluronic acid (HA) can further enhance the migration capabilities of MSCs [21,22]. Moreover, natural cell membranes retain specific proteins from the cell surface, reducing immunogenicity and displaying strong biocompatibility. PLGA, a biodegradable polymer that possesses advantages, such as biocompatibility and biodegradability, offers a passive mechanism that can enable site-specific drug release, thereby reducing systemic effects. PLGA is widely used to prepare nanoparticles to encapsulate hydrophilic and hydrophobic therapeutic agents [23]. We designed a strategy for coating the surface of the core of PLGA nanoparticle with MSC membranes. The surface functionalization of MSC membranes endows PLGA nanoparticles (PLGA/MSCs) with intrinsic inflammation-targeting features and enables them to exhibit active and passive targeting effects to achieve effective delivery of drugs against ALI.

Cryptotanshinone (CPT) is an active lipophilic compound derived from the plant *Salvia miltiorrhiza* Bunge, known for its remarkable anti-inflammatory, hepatoprotective, and antioxidant properties. Nagappan et al. [24] demonstrated that CPT exerts its hepatoprotective effects against ethanol-induced liver injury by activating the AMPK/SIRT1 and Nrf2 pathways while simultaneously inhibiting CYP2E1. This phenomenon leads to the suppression of fat accumulation, oxidative stress, and inflammatory responses. Despite these promising attributes, pharmacokinetic studies have revealed that CPT suffers from low absorption rates and limited bioavailability, thereby presenting challenges to its clinical application [25].

**Figure 1:**
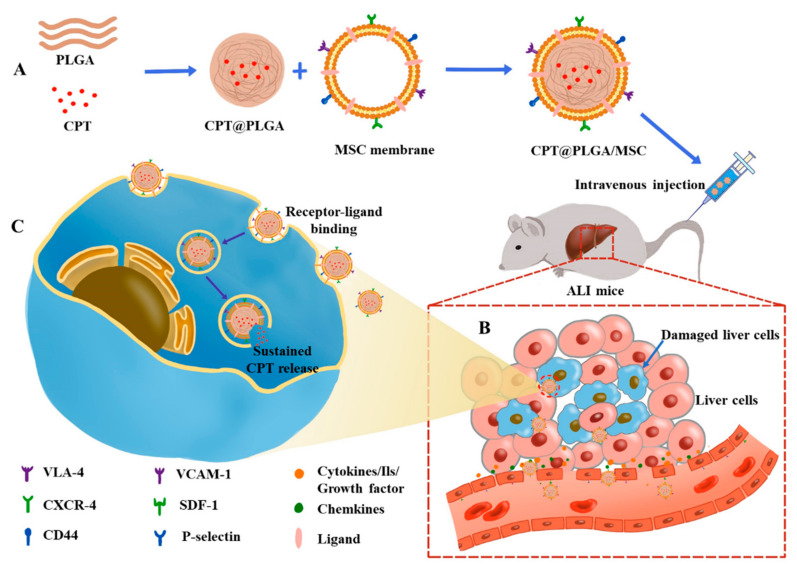
Overall strategy of CPT@PLGA/MSCs targeting ALI: (**A**) Construction of biomimetic nanoparticles: CPT@PLGA/MSC. (**B**) Schematic of the homing/migration mechanism: CPT@PLGA/MSCs are attracted by various chemotactic factors, migrating from distant areas to the vicinity of the lesion, followed by adhesion to endothelial cells highly expressing adhesion molecules and integrins in the lesion area. After adhesion, CPT@PLGA/MSCs migrate through the endothelial layer into the lesion site. (**C**) Hepatic cell internalization of CPT@PLGA/MSC: CPT@PLGA/MSCs enter hepatic cells through receptor-mediated endocytosis, releasing CPT into the cytoplasm to exert its therapeutic effect.

In light of these limitations, the design of an efficient drug delivery system for CPT to enhance its bioavailability is crucially needed. Our research led to the development of novel biomimetic nanoparticles wherein CPT is encapsulated within PLGA nanoparticles as the core and is surrounded by MSC membranes overexpressing CD44 (CPT@PLGA/MSCs). This innovative nanocarrier system is specifically designed for the targeted therapy of ALI cases and thus presents a promising and novel treatment strategy for liver-related diseases (Figure 1). By utilizing this strategy, the biomimetic drug delivery system enables the targeted delivery of CPT to the injured liver, thereby maximizing its therapeutic efficacy and overcoming its pharmacokinetic limitations. This cutting-edge nanomedicine represents an important advancement in liver-targeted treatments and offers potential benefits to patients suffering from ALI. Utilizing this innovative approach could pave the way for effective and precise therapeutic interventions in the field of liver-related disorders.

## 2. Materials and Methods

### 2.1. Materials

Dulbecco’s Modified Eagle Medium High-glucose, Dulbecco’s Modified Eagle Medium low-glucose, bicinchoninic acid (BCA) protein quantitative kit, 4′,6-diamidino-2-phenylindole (DAPI), and phosphate-buffered saline (PBS) were purchased from Guangzhou Sopo Biological Technology Co., Ltd. (Guangzhou, China). Fetal bovine serum (FBS) was obtained from Gibco (Grand Island, NY, USA). Cryptotanshinone (purity ≥ 97%) was provided by Nanjing Dilger Medical Technology Co., Ltd. (Nanjing, China). Alanine aminotransferase (ALT), Aspartate aminotransferase (AST), alkaline phosphatase (AKP), malondialdehyde (MDA), superoxide dismutase (SOD), reduced glutathione (GSH), catalase (CAT, creatinine (CRE), and urea nitrogen (BUN) were purchased from Nanjing Jiancheng Bioengineering Institute. The tumor necrosis factor (TNF-α), interleukin-1β (IL-1β), and interleukin-6 (IL-6) enzyme-linked immunosorbent assay (ELISA) kits were purchased from Elabscience Biotechnology Co., Ltd. (Wuhan, China). Carbon tetrachloride (CCl_4_) and olive oil were purchased from Shanghai Macklin Biochemical Co., Ltd. (Shanghai, China). Hydrophilic polyvinylidene fluoride (PVDF) membranes and supersensitive electrogenerated chemiluminescence (ECL) luminescence reagent were purchased from Solarbio Science & Technology Co., Ltd. (Beijing, China). Antibodies, including PE-antiCD29, FITC-anti-CD90, FITC-anti-CD45, FITC-anti-CD44, and PE-anti-CD34, were obtained from BD Pharmingen (San Diego, CA, USA). CD44 antibodies were obtained from Affinity Biosciences Pty Ltd. (Melbourne, Australia). Monocyte chemoattractant protein-1 (MCP-1) was purchased form Proteintech Group, Inc. (Chicago, IL, USA).

### 2.2. Cell Culture and Animals

LO2 cells were purchased from the American Type Culture Collection (ATCC, Manassas, VA, USA). SD rats (male, 20 day) and ICR mice (male, 20 ± 2 g) were purchased from Chengdu Dossy Experimental Animals Co., Ltd. (Chengdu, China). LO2 cells were cultured in high-glucose DMEM containing 10% FBS. As previously reported, MSCs were extracted from the bone marrow of SD rats [26] and maintained in low-glucose DMEM containing 10% FBS. Cells between 3 and 5 passages were used. All cells were cultured in a 5% CO_2_ incubator at 37 °C. Flow cytometry was employed to characterize and analyze MSCs [27,28]. At passage 3, the surface marker proteins of MSCs were identified through analysis with Flow BD FACSC Anto II (BD, San Jose, CA, USA) and CD44, CD34, CD45, CD90, and CD29 antibodies. MSCs were stimulated with HA (30 μg/mL) for 30 min in culture medium and then collected and stained with an FITC-conjugated anti-CD44 antibody. The CD44 cell surface was subjected to flow cytometry.

### 2.3. Migration Assay

Transwell chambers and 8 μm pore polycarbonate filters (LABSELECT, Guangzhou, China) were used to monitor cell migration in vitro [29]. Prior to the experiment, cells were collected and resuspended in low-glucose DMEM without FBS. MSCs (5 × 10^4^ cells/well) were placed in the upper chamber of a Transwell unit. The lower chamber of the Transwell unit contained 10% FBS and low-glycemic DMEM with or without 30 μg/mL HA. MSCs were allowed to migrate for 48 h through the membrane pores. At the end of migration, they were fixed with 4% paraformaldehyde and stained with crystal violet. Non-migrated cells on the upper surface of the polycarbonate membrane were gently removed, and cells in randomly selected fields of view were observed and counted with an inverted microscope (ix73, Olympus, Tokyo, Japan).

### 2.4. Separation of MSC Membranes

MSCs were incubated with HA in an incubator for 30 min when 80–90% confluent [21,30]. Subsequently, they were washed three times, harvested through centrifugation (1200 rpm, 5 min), and resuspended in hypotonic lysate (containing 0.35 M sucrose, 0.001 M MgCl_2_, 0.01 M Tris, and 10 μg/mL DNase; pH 7.4). Cells were crushed using an ultrasonic cell crusher for 2 min (20% amplitude, T_on_ = 3 s, T_off_ = 7 s), and the resulting suspension was then centrifuged at 4000 rpm at 4 °C for 10 min. The resulting supernatant was further centrifuged at 15,000 rpm for 30 min at 4 °C to obtain pellet membranes. MSC membranes were resuspended in PBS and stored at −80 °C for further experimentation.

### 2.5. Preparation and Characterization of the Nanoparticles

PLGA nanoparticles were prepared by using the emulsion solvent evaporation method described in previous laboratory research. In brief, a homogeneous mixture of 1 mL of PLGA (20 mg/mL, CH_2_Cl_2_) and 0.2 mL of CPT (6.7 mg/mL, CH_2_Cl_2_) was slowly added to 5 mL of 0.1% polyvinyl alcohol solution. The mixture was then emulsified with an ultrasonic cell disruptor for 5 min (35% amplitude, T_on_ = 5 s, T_off_ = 5 s). Dichloromethane was then removed using a rotary evaporator. PLGA nanoparticles loaded with CPT (CPT@PLGA) were collected via centrifugation at 15,000 rpm for 30 min at 4 °C, washed, and sequentially passed through 400 and 200 nm polycarbonate filter membranes using a mini extruder (Nuclepore, Pleasanton, CA, USA). The mass ratio of membrane protein to CPT@PLGA was 1:5 [31]. The MSC membrane solution was mixed with CPT@PLGA and sequentially passed through 400 and 200 nm polycarbonate filter membranes using a mini extruder to integrate MSC membranes onto the surfaces of PLGA nanoparticles. The mixture was centrifuged at 15,000 rpm for 30 min at 4 °C, and the resulting precipitate was denoted as MSC membrane-coated PLGA nanoparticles (CPT@PLGA/MSC).

Transmission electron microscopy (TEM, JEM-1230, Tokyo, Japan) was utilized to examine the morphology of the nanoparticles. The size distribution, zeta potential, and polydispersity index (PDI) of the nanoparticles were assessed using a Malvern Zetasizer system (Nano ZS, Malvern Instruments, Malvern, UK). CPT@PLGA/MSCs were preserved in PBS at 4 °C to monitor their size, zeta potential, and PDI every day for 7 days.

A precise volume of 1 mL of the prepared CPT@PLGA/MSC solution was mixed with 4 mL of acetonitrile to examine encapsulation efficiency and drug loading content. The mixture was sonicated in a water bath for 10 min to break the structure of the nanoparticles and release the drug. The high-performance liquid chromatography (HPLC, Agilent, Santa Clara, CA, USA) methodology for CPT was established (Supplementary File Figure S1). The conditions were as follows: the chromatographic column used was an Agilent-C18 (4.6 × 100 mm, 2.7 µm); the mobile phase comprised acetonitrile and 0.1% phosphate water in a ratio of 59:41 (*v*/*v*); the detection wavelength was set at 270 nm; the injection volume was 10 μL; the flow rate was set at 1.0 mL/min; and the column temperature was maintained at 35 °C. The CPT content in the solution was quantified using HPLC and was denoted as W_1_. Subsequently, 1 mL of the CPT@PLGA solution was accurately transferred to a 10 mL EP tube and then centrifuged at 4 °C and 15,000 rpm for 30 min. The supernatant was discarded, and 5 mL of acetonitrile was added to disrupt the particles. The resulting solution was sonicated for 10 min in a water bath. The CPT content was measured through HPLC and recorded as W_2._ Encapsulation efficiency (EE) was calculated as W_2_/W_1_ × 100%.

For the determination of drug loading, 1 mL of freshly prepared CPT@PLGA nanoparticle solution was precisely extracted and centrifuged. The supernatant was discarded, and the obtained pellet was resuspended in 1 mL of ultrapure water and subsequently freeze-dried. The mass of the container was measured in triplicate before freeze drying, and the average value was obtained. After freeze drying, the dried powder was weighed, and the mass of the container was subtracted. The mass of the freeze-dried nanoparticle powder was recorded as W_3_. Subsequently, the dried powder was reconstituted with 1 mL of acetonitrile for sonication, and CPT content was determined through HPLC and denoted as W_4_. Drug loading capacity (LC) was calculated as W_4_/W_3_ × 100%.

MSC membranes and PLGA/MSC were subjected to sodium dodecyl–sulfate polyacrylamide gel electrophoresis (SDS–PAGE). Cell lysate containing phenylmethanesulfonyl fluoride protease inhibitor at a ratio of 100:1 was added to the samples, which were lysed for 30 min in an ice bath and centrifuged at 12,000 rpm for 10 min at 4 °C. Protein content was measured using a BCA assay kit. Samples were loaded onto 12% Tris gels and electrophoresed using a Bio-Rad electrophoresis system (Hercules, CA, USA). After electrophoresis, the protein gel was stained with Coomassie brilliant blue, decolorized, and imaged using a Bio-Rad imaging system.

Western blot analysis was performed to detect the cell membrane protein marker CD44. After electrophoresis, proteins were subsequently transferred onto a PVDF membrane using a semidry transfer method. The PVDF membrane was blocked with 5% skimmed milk in TBST for 1 h and incubated with primary antibodies overnight at 4 °C. Subsequently, it was washed with TBST and incubated at room temperature with an HRP-conjugated secondary antibody for 1 h. ECL chemiluminescence was applied, and protein bands were visualized using a Bio-Rad protein gel imaging system.

### 2.6. Drug Release In Vitro

A total of 1 mL of CPT@PLGA or CPT@PLGA/MSC solution was dialyzed against 30 mL of PBS (pH 7.4) containing 0.5% Tween 80 using a dialysis bag (MWCO: 8000–14,000) with stirring at 100 rpm and 37 °C to simulate in vitro drug release [32]. Subsequently, 1 mL of the sample was collected and replenished with an equal volume of buffer at 0, 1, 2, 4, 8, 12, and 24 h. CPT content was determined through liquid chromatography–mass spectrometry (LC–MS, LCMS-2020, Shimadzu, Kyoto, Japan). The cumulative release percentage of CPT was calculated using the following formula:

$$\mathrm{Cumulative}\;\mathrm{release}\;\mathrm{rate}(\%)=\frac{\sum_{1}^{n-1}\left(\mathrm{Cp}\;\times \;\mathrm{Vp}\right)+\mathrm{Cn}\;\times \;V}{m}\;\times \;100\%$$

C_p_: the concentration of CPT at the points prior to n; V_p_: the volume of the collections; C_n_: the concentration of CPT at collection point n; V: the initial total volume; m: total content of CPT. 

### 2.7. Cell Viability

LO2 cells were used as cell models in vitro, and cell viability in the presence of the nanocarriers and drugs was evaluated via CCK-8 assay. The LO2 cell suspension was adjusted to a concentration of 1 × 10^5^ cells/mL and seeded into 96-well plates at a density of 100 μL/well for 24 h. The cells were then treated with free CPT, CPT@PLGA, and CPT@PLGA/MSC with the equivalent CPT concentrations of 2.5, 5, 10, 20, and 40 μg/mL. The control group comprised cells in drug-free culture medium, whereas the blank group included cells in cell- and drug-free culture medium. Next, 10 μL of CCK-8 solution was added to each well after 24 h of incubation. Subsequently, absorbance at 450 nm was measured using a microplate reader (LYT5M, BioTek, Winooski, VT, USA). Cell viability (%) = [(OD experimental group − OD blank group)/(OD control group − OD blank group)] × 100%.

### 2.8. Cellular Uptake

In the cellular uptake experiments, LO2 cells were seeded in six-well plates at a density of 1 × 10^5^ cells/well for 24 h. CPT was replaced by DIL (λex/λem = 549/565 nm, red fluorescent dye, 10 μM) and DIL@PLGA/MSC was prepared according to Method 2.5, which was then added into six-well plates and incubated for 1, 2, and 4 h. Subsequently, the cells were washed with cold PBS three times to remove free vehicles. The cells were fixed with 4% neutral paraformaldehyde solution at room temperature and counterstained with DAPI before being imaged with an inverted fluorescence microscope. MSC membranes were incubated with DIO (λ_ex_/λ_em_ = 484/501 nm, green fluorescent dye 10 μM) on a shaker at 37 °C for 1 h to label MSC membranes. DIO-labeled MSC membranes and DIL-labeled PLGA were utilized to prepare dual-labeled DIL@PLGA/MSC^DIO^ to further confirm that the MSC membranes had successfully coated the surfaces of PLGA nanoparticles. LO2 cells were incubated for 4 h. Subsequently, an inverted fluorescence microscope was used to observe the colocalization of fluorescence after fixation and DAPI staining.

### 2.9. Anti-Inflammatory and Antioxidant Efficacy In Vitro

LO2 cells (1 × 10^5^ cells/well) were seeded into 24-well plates and maintained in a 5% CO2 incubator at 37 °C for 24 h to allow cell adhesion. The old medium was discarded and freshly prepared DMEM solution (1 mL) containing H_2_O_2_ (30 µg/mL) and drug-carrying nanoparticles with the CPT contents of 20 μg/mL were added into the 24-well plates. Cells and the supernatant were collected after 24 h to assess the levels of SOD, CAT, TNF-α, IL-6, and IL-1β in accordance with the kit requirements [33].

### 2.10. Establishment of ALI Model

All experimental animals were 20 ± 2 g male ICR mice unless otherwise noted. ALI models were established at 6 h after a single intraperitoneal injection of 10% 2.5 mL/kg CCl_4_ [34] (diluted to 10% in olive oil and filtered by using a 0.22 μm filter before administration). Animal experiments were conducted in accordance with the National Act on the Use of Experimental Animals (Chengdu, China) and approved by the Sichuan Committee on Laboratory Animals (approval number: SYXK2019-216).

### 2.11. Imaging In Vivo

PLGA and PLGA/MSC were loaded with Cy5.5 according to Method 2.5, and a PLGA/MSC group without HA pretreatment was established. Each group contained an equal amount of Cy5.5 fluorescent dye. The nanoparticles were intravenously injected into ALI mice to validate the liver-targeting ability of the nanoparticles. The ALI mice in the blank group were injected with the same volume of PBS. After 8 h, the mice were euthanized, and their major organs were separated for ex vivo imaging by using an in vivo imaging system (IVIS Spectrum, Maestro, Trenton, NJ, USA). Subsequently, liver tissue was promptly subjected to frozen sectioning, and the distribution of fluorescence was observed.

### 2.12. Therapeutic Effect In Vivo

The mice were randomized into the following treatment groups: (1) normal group: no ALI induction or therapy; (2) model group: ALI induction + tail vein injection of PBS; (3) free CPT treatment group: ALI + tail vein injection of CPT; (4) CPT@PLGA treatment group: ALI + tail vein injection of CPT@PLGA; and (5) CPT@PLGA/MSC treatment group: ALI + tail vein injection of CPT@PLGA/MSC. The CPT contents were all 10 mg/kg. The same treatment protocol was repeated on day 3. Blood samples were collected from the orbital cavities of the mice on day 4. After blood sample collection, the mice were euthanized through cervical dislocation. Liver tissue was harvested, and part of it was fixed for histological examination through histopathological hematoxylin–eosin (H&E) staining and immunohistochemistry (IHC). Serum and liver tissue homogenates were subjected to analysis.

### 2.13. In Vivo Biosafety Evaluation

Healthy mice were intravenously injected with PBS, CPT@PLGA, CPT@PLGA/MSC, or an equivalent dose of free CPT (10 mg/kg) on days 1 and 3 to evaluate the toxicity of the drug and carrier. Blood was collected, and all mice were sacrificed on day 4. Major organs were collected and weighed. All collected tissues were fixed with 4% paraformaldehyde solution for subsequent H&E staining. The serum levels of ALT, AST, LDH, CRE, and BUN were measured using biochemical assay kits. 

### 2.14. Statistical Analysis

All results presented in this work were expressed as means ± standard deviation (SD). Data analysis was conducted and graphs were drawn by GraphPad Prism 8.0.2 (GraphPad Software, La Jolla, CA, USA). Comparisons among three or more groups were performed using one-way ANOVA. * *p* < 0.05, ** *p* < 0.01, *** *p* < 0.001, and **** *p* < 0.0001 indicated statistical difference, and ns meant no significant difference.

## 3. Results

### 3.1. Identification of MSCs

**Figure 2:**
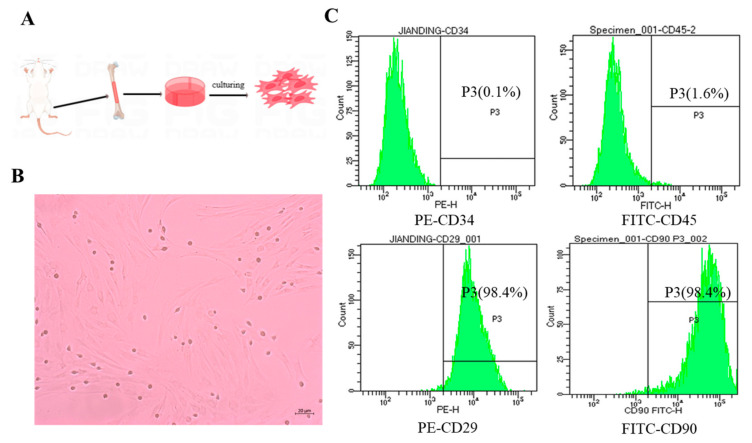
Extraction and characterization of MSCs. (**A**) Schematic illustration of MSC extraction from bone marrow. (**B**) The shape of cultured MSCs (scale bar: 20 μm). (**C**) Expression of MSC surface markers identified using flow cytometry.

A schematic of the extraction of MSCs from rat bone marrow is illustrated in Figure 2A. The cultured MSCs had a spindle-shaped, fibroblast-like morphology with clearly delineated cell margins (Figure 2B). Flow cytometry revealed that, on the surface of the MSCs, the positive expression rates of the MSC-positive markers CD90 and CD29 exceeded 95%, whereas those of the MSC-negative markers, CD34 and CD45, were less than 2% (Figure 2C), indicating that the cells met the international definition of MSCs [35].

### 3.2. Migration Assay and HA Affects CD44 Expression

**Figure 3:**
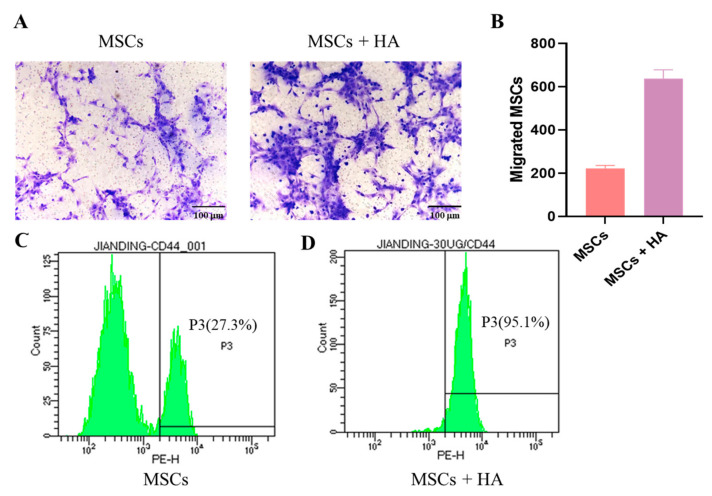
Homing potential of MSCs exposed to HA and the effects of HA on CD44 expression. (**A**) Migrated cells were stained with 0.2% crystal violet (×200, scale bar:100 μm). (**B**) Quantification of migrated cells. Data are presented as the mean number of migrated cells ± SD (n = 3). (**C**,**D**) The expression of CD44 on MSC surfaces with or without HA pretreatment.

HA has been demonstrated to possess chemotactic properties for CD44-MSCs [30,36]. A Transwell experiment was conducted with the addition of 30 μg/mL HA in the lower chamber to investigate MSC migration in vitro. The control group was not treated with HA. The results revealed significant alterations in the quality and quantity of cell migration when HA was introduced (Figure 3A,B). Notably, treatment with 30 μg/mL HA resulted in a remarkable 2.86-fold increase in the in vitro migration capacity of MSCs. Flow cytometry results revealed that the positive expression rates of marker CD44 on MSC membranes increased from 27.3% to 95.1% after stimulation with 30 μg/mL HA (Figure 3C,D). Consequently, subsequent experiments were conducted by employing an HA concentration of 30 μg/mL.

### 3.3. Characterization of Nanoparticles

**Figure 4:**
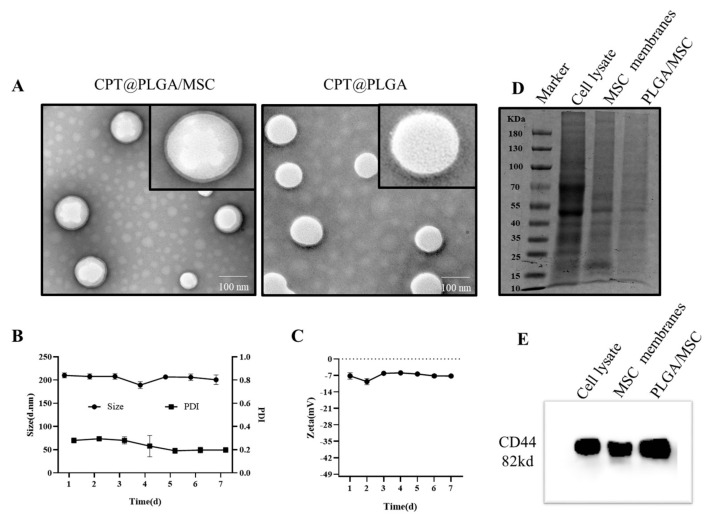
Characterization of the nanoparticles. (**A**) TEM images of CPT@PLGA and CPT@PLGA/MSC (scale bar:100 nm). (**B**) The size distribution and PDI of CPT@PLGA/MSC were examined using a Malvern Zetasizer. All the data are presented as mean ± SD (n = 3). (**C**) The zeta potential of CPT@PLGA/MSC was determined by a Malvern Zetasizer. All the data are presented as mean ± SD (n = 3). (**D**) SDS-PAGE analysis of the retention protein. (**E**) Western blotting for analyzing the surface marker proteins.

TEM imaging revealed that CPT@PLGA and CPT@PLGA/MSC had uniform spherical nanostructures, confirming that MSC membranes coated CPT@PLGA/MSC but not CPT@PLGA (Figure 4A). Dynamic light scattering analysis revealed that CPT@PLGA had an initial size of 183.9 ± 4.5 nm, a PDI of 0.225 ± 0.048, and a zeta potential of -21.7 ± 0.7 mV (Table 1). Upon membrane modification, the size of the CPT@PLGA/MSC increased to 204.3 ± 4.5 nm, with a PDI of 0.270 ± 0.028, and the zeta potential shifted to -9.6 ± 0.9 mV (Table 1), close to that of MSC membrane vesicles (-10.5 ± 0.9 mV). The sizes of PLGA nanoparticles increased by 20 nm, which was close to the thickness of cell membranes, and suggested that PLGA nanoparticles were successfully coated with MSC membranes. The negative charge on the surfaces of CPT@PLGA/MSC provided protection against nonspecific protein adsorption, ensuring their stability in the bloodstream [37]. 

SDS–PAGE and Western blot analysis were performed to examine and compare the protein expression patterns of whole cells, purified MSC membranes, and PLGA/MSC. As shown in Figure 4D, the protein profiles of PLGA/MSC and purified plasma membranes exhibited similarities, distinguishing them from the protein profile of the whole cell lysate. In addition, proteins located on MSC membranes, particularly CD44 [38], possess a pivotal role in targeting inflammatory processes. The presence of the membrane protein CD44 on PLGA/MSC was confirmed through Western blot analysis (Figure 4E). This result indicated the successful transfer and preservation of proteins on the surfaces of PLGA nanoparticles [39]. 

Next, the storage stability and loading efficiency of CPT@PLGA/MSC were evaluated. The slight changes in their size, PDI, and zeta potential indicated that CPT@PLGA/MSC was relatively stable and had good stability in PBS storage solution (Figure 4B,C). The EE and LC of CPT@PLGA were 93.74 ± 1.07% and 5.74 ± 0.45%, respectively. For CPT@PLGA/MSC, the EE and LC were 88.16 ± 1.77% and 4.76 ± 0.11%, respectively.

### 3.4. Drug Release

**Figure 5:**
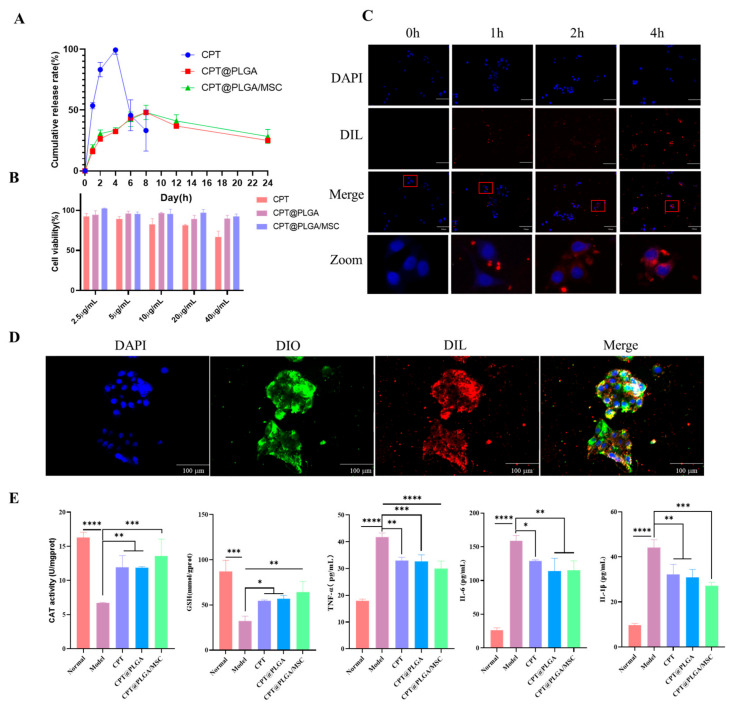
The drug release profile and in vitro study in cells. (**A**) In vitro CPT cumulative release profiles in water. All the data are presented as mean ± SD (n = 3). (**B**) Cytotoxicity of CPT at concentrations in the range of 2.5–40 μg/mL. All the data are presented as mean ± SD (n = 3). (**C**) Images of LO2 incubated with DIL@PLGA/MSC for 0, 1, 2, and 4 h; blue and red colors represent DAPI and DIL, respectively (×200, scale bar: 100 μm). (**D**) Colocalization of MSC membranes (green dots labeled by DIO) and PLGA nanoparticles (red dots labeled by DIL) after incubation with LO2 for 4 h (×200, scale bar: 100 μm). (**E**) ELISA and oxidant stress results indicating the levels of CAT, GSH, IL-6, IL-1β, and TNF-α in CPT@PLGA/MSC-treated and H_2_O_2_-stimulated LO2 at 24 h. All the data are presented as mean ± SD (n = 3), * *p* < 0.05, ** *p* < 0.01, *** *p* < 0.001, and **** *p* < 0.0001.

The release of CPT in CPT@PLGA/MSC and CPT@PLGA exhibited a slow pattern, with the highest drug release rates observed at 8 h, reaching 48.01 ± 5.83% and 48.02 ± 2.09%, respectively (Figure 5A). Compared to free drug, CPT loaded onto the nano-carriers demonstrated favorable sustained release characteristics, suggesting that these sustained release properties may contribute to enhancing the therapeutic efficacy of the drug.

### 3.5. Cytotoxicity

Cell viability after nanoparticle administration was studied using a CCK-8 assay. The cell survival rates after 24 h of treatment with varying concentrations of free CPT decreased with the increase in free CPT concentration to 92.63 ± 2.85%, 89.02 ± 2.65%, 82.31 ± 6.06%, 81.18 ± 1.11%, and 66.71 ± 6.12% (Figure 5B). The results showed that free CPT had a cytotoxic effect when its concentration was excessively high. By contrast, LO2 cells showed survival rates that remained above 85% after 24 h of coincubation with CPT@PLGA and CPT@PLGA/MSC at the CPT concentration of 2.5–40 μg/mL. The absence of considerable cytotoxicity indicated that, within a certain concentration range, the carrier material had good biocompatibility with cells. The pretreatment of liver cells with 40 μM of CPT inhibited reactions, such as lactate dehydrogenase leakage and GSH depletion, induced by TBH or GalN in vitro [40]. Therefore, in consideration of the comprehensive cytotoxicity results, an in vitro experimental dose of 20 μg/mL CPT was selected. 

### 3.6. Cellular Uptake and Colocalization In Vitro

Effective drug absorption by target cells is a prerequisite for drugs to exert their therapeutic efficacy. In this study, the cellular uptake profile of DIL@PLGA/MSC was determined. Cells were exposed to fluorescent nanoparticles for 1, 2, and 4 h. Fluorescence imaging revealed that nanoparticles began to approach cells at 1 h, and a substantial portion of nanoparticles was observed near cell nuclei at 4 h. This observation strongly suggested that PLGA/MSC nanoparticles can be effectively internalized by LO2 cells (Figure 5C). Furthermore, microscopy observation revealed that the nanoparticle core and MSC membranes mostly colocalized, demonstrating the successful coating of MSC membranes on the surfaces of PLGA nanoparticles (Figure 5D).

### 3.7. Pharmacodynamics In Vitro

The therapeutic effect of nanoparticles on H_2_O_2_-induced damage in LO2 cells was assessed by using oxidative stress assay and ELISA kits. The results showed that, in LO2 cells, the levels of oxidative stress and inflammation significantly increased after H_2_O_2_ treatment, confirming the successful establishment of the LO2 cell oxidative stress model. In addition, CPT@PLGA/MSCs generated a larger therapeutic benefit in reducing the levels of oxidative stress and inflammation than free CPT and CPT@PLGA (Figure 5E), contributing to the subsequent in vivo pharmacodynamics experiments.

### 3.8. Liver Targeting

**Figure 6:**
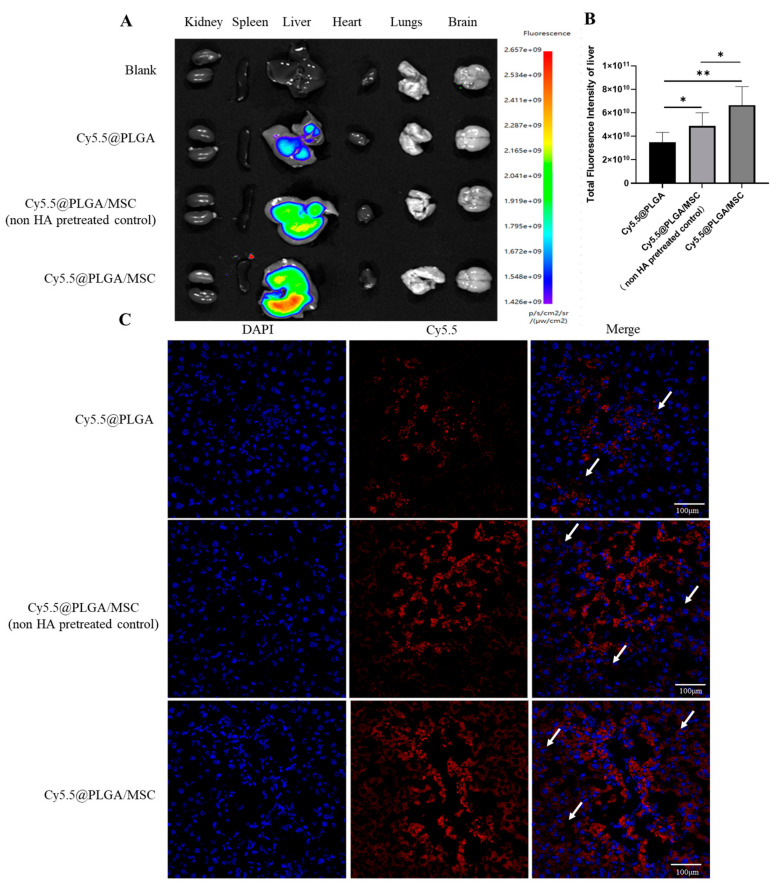
Tissue distributions of biomimetic nanoparticle. (**A**) Ex vivo images of mouse organs at 8 h. All the data are presented as mean ± SD (n = 6). (**B**) Semi-quantitative results of liver fluorescence. All the data are presented as mean ± SD (n = 3). (**C**) Specific distribution in the liver (×200, scale bar: 100 μm). The arrows represent the transport of nanoparticles to HSC cells. * *p* < 0.05, ** *p* < 0.01.

In vivo imaging provides a comprehensive visual method for studying nanoparticle distribution in mice. Mice were euthanized at particular time points, and their organs were dissected and then imaged using an advanced imaging system. After coating with MSC membranes, the nanoparticles exhibited a significantly increased targeting ability toward the liver. The fluorescence intensity in the liver was notably higher in the Cy5.5@PLGA/MSC group compared to the Cy5.5@PLGA/MSC group without HA pretreatment (Figure 6A). The liver tissues of mice were prepared in frozen sections, and their fluorescence distribution was observed under a fluorescence microscope to gain further insights into the specific distribution of nanoparticles within the liver. The results revealed that the fluorescent-labeled nanoparticles predominantly accumulated in inflamed liver sinusoids, with the Cy5.5@PLGA/MSC group exhibiting the highest quantity of fluorescent nanoparticles among the three groups (Figure 6C). These findings were in line with the ex vivo imaging results. Collectively, the data strongly indicated that MSC membrane-coated PLGA nanoparticles effectively enhanced drug uptake efficiency in the liver and possessed exceptional liver-targeting capabilities. The targeting ability appeared to be enhanced after HA pretreatment. The damage-targeting effect of PLGA/MSC can be likely attributed to the expression of numerous adhesion molecules and chemokine receptors that can bind to their corresponding adhesion molecules and chemokines in damaged tissues [38].

### 3.9. Therapy Efficacy In Vivo

**Figure 7:**
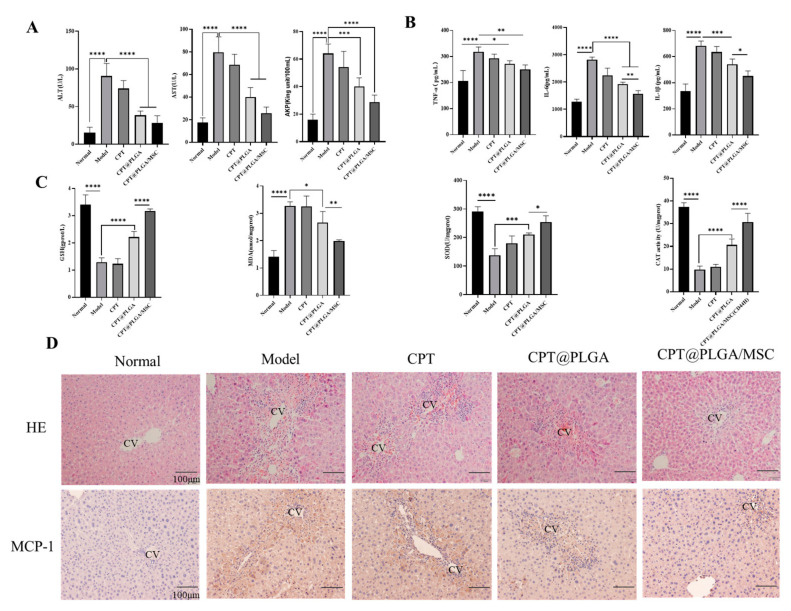
CPT@PLGA/MSCs were used to treat acute liver injury in mice. (**A**) The level of AST, ALT, and AKP in the serum. All the data are presented as mean ± SD (n = 5). (**B**) Inflammatory factor (TNF-α, IL-6, and IL-1β) levels in liver tissues. All the data are presented as mean ± SD (n = 5). (**C**) The oxidative stress levels (SOD, GSH, MDA, and CAT) in liver tissues. All the data are presented as mean ± SD (n = 5). (**D**) HE staining of liver tissue sections (×200, scale bar: 100 μm). Representative images of MCP-1 immuno-stained liver tissue sections (×200, scale bar: 100 μm), CV: central vein. * *p* < 0.05, ** *p* < 0.01, *** *p* < 0.001, and **** *p* < 0.0001.

ALP, ALT, and AST are excellent biomarkers for the evaluation of liver functions. Figure 7A shows that the CCl_4_ group exhibited significantly increased ALT, AST, and ALP production compared to the normal group, indicating that the ALI model was successfully established. The nanoparticle-treated groups showed a significant reduction in serum ALT, AST, and AKP levels compared with the model group (Figure 7A). Notably, the CPT@PLGA/MSC group exhibited greater therapeutic benefits than the other groups. The SOD, CAT, GSH, and MDA levels in liver tissue were measured to assess the potential antioxidant effect of the drugs. The results revealed that the nanoparticle-treated group demonstrated a notable decrease in liver oxidative stress levels compared to the model group (Figure 7C). Similarly, the CPT@PLGA/MSC group displayed superior therapeutic effects in this regard. Treatment with CPT@PLGA/MSC effectively reduced the levels of proinflammatory cytokines, including IL-6, IL-1β, and TNF-α, in the liver (Figure 7B). This outcome strongly suggested the anti-inflammatory properties of CPT@PLGA/MSC and may imply that CPT@PLGA therapy could reduce CCl_4_-induced hepatic damage by antioxidants and anti-inflammatory drugs.

H&E staining and IHC were performed to observe the histopathological changes and specific protein (MCP-1) expression in the mouse liver. The H&E staining results presented in Figure 7D revealed the protective effect of the nanoparticle formulation on liver morphology. In the control group, the hepatic lobular structure and central vein region appeared normal, indicating a healthy liver. However, a disruption in the hepatic lobular structure and the presence of cellular necrosis were observed in the model group, indicating liver damage. Treatment with free CPT and CPT@PLGA partially restored the hepatic lobular structure, although considerable inflammatory cell infiltration remained in the central vein region, suggesting ongoing inflammation. By contrast, the liver morphology of mice treated with CPT@PLGA/MSC closely resembled that of the control group, indicating the effective restoration of liver structure. MCP-1, which is produced primarily by activated monocytes and one of the most important bioactive lysophospholipids, has been proven to be markedly increased in injured livers [41,42]. The liver tissue of each treatment group was subjected to IHC staining for MCP-1. Compared to the control group, the model group exhibited a large area of positive staining in the sinusoidal region of liver tissue, indicating increased MCP-1 expression due to the intraperitoneal injection of CCl_4_, which is known to induce liver damage. Meanwhile, all treatment groups showed a reduction in MCP-1 expression (Figure 7D). Among the groups, the CPT@PLGA/MSC group demonstrated the most pronounced effect and closely resembled the control group in terms of MCP-1 expression. This observation suggested that the homing effect of MSCs resulted in the increased accumulation of nanoparticles in the damage region, leading to high drug concentration and, consequently, the significant downregulation of MCP-1 expression.

### 3.10. In Vivo Biosafety Evaluation

**Figure 8:**
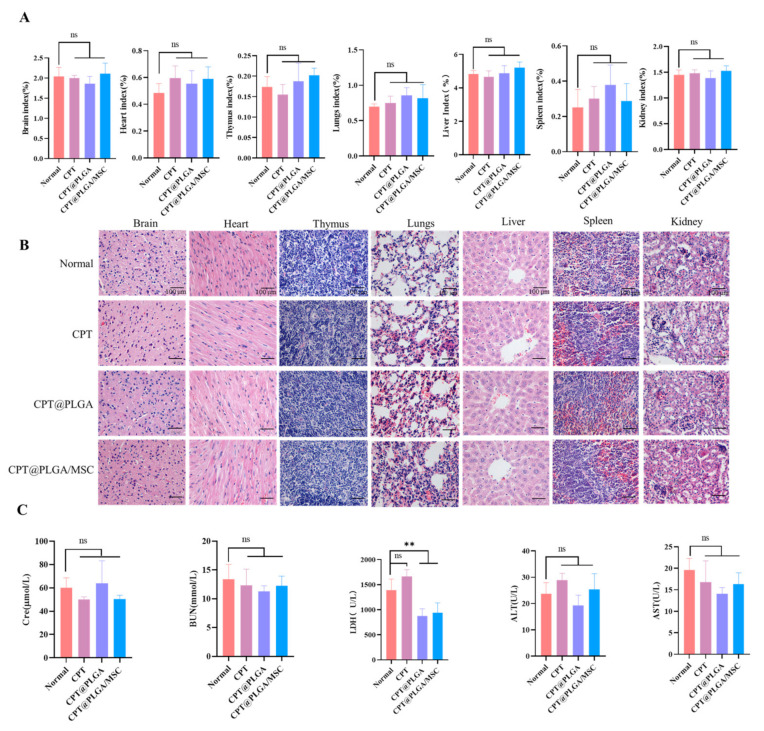
CPT@PLGA/MSC exhibited an excellent safety profile in vivo. (**A**) Organ index after treatment with CPT, CPT@PLGA, and CPT@PLGA/MSC. All the data are presented as mean ± SD (n = 5). (**B**) The HE staining of mouse brain, heart, thymus, lung, liver, spleen, and kidney tissue sections (×400, scale bar: 100 μm). (**C**) Serum levels of BUN, Cre, ALT, AST, and LDH. All the data are presented as mean ± SD (n = 5). ns indicated no significance; ** *p* < 0.01.

None of the three treatment groups showed any significant changes in visceral index (Figure 8A). They did not exhibit notable abnormalities in the levels of biochemical indicators, including ALT, AST, Cre, and BUN. In addition, the reduction of LDH was not clinically significant. (Figure 8C). Correspondingly, the H&E staining images revealed no apparent pathological findings in the treatment groups relative to the normal group (Figure 8B). 

## 4. Discussion

A strategy to overcome the challenges of poor drug absorption and limited bioavailability in the body involves drug delivery with nanoparticles. However, the utilization of nanomaterials in drug delivery systems has certain constraints, including their susceptibility to swift clearance by the immune system and their lack of precise cell-targeting capabilities [43,44,45]. Researchers have discovered that encapsulating cores of nanometer-sized particles by biomimetic coatings composed of cell membranes is a solution for mitigating the rapid immune system clearance of nanomaterials [46]. Biomimetic coating technology maintains membrane proteins associated with intercellular communication and immune modulation, conferring the biomimetic nanoparticles with the physicochemical characteristics of the source cells and allowing them to retain their distinct biological functionalities [47,48]. The regenerative property of MSCs is primarily attributed to their capability of homing back to sites of injury or inflammation. This homing ability has positioned MSC membranes as a potential candidate for targeted drug delivery in cases of ALI [48,49,50]. CD44 [51], a multifunctional receptor on the surfaces of MSCs, plays a pivotal role in steering MSCs to sites of injury or inflammation [52]. The increase in the levels of its specific ligand, HA, is typically associated with inflammation, and the interaction between CD44 and HA plays a dominant role in the migration of exogenous MSCs toward damaged tissues [53]. These findings led the authors to formulate strategies aiming to amplify and enhance the migration, localization, and retention abilities and therapeutic efficacy of MSCs. Notably, the role of the CD44 receptor and its ligand HA in recruiting MSCs has been well investigated using rodent models with renal failure [29]. In these cases, culture media were supplemented with soluble HA, and HA was found to enhance the chemotactic properties of MSCs in vitro and in vivo. An interaction between HA (0.5–1 mg/mL) and CD44 induced the transient but significant upregulation of CD44 on MSC membranes after 24 h, as confirmed in an LPS-induced inflammation mouse ear model [21]. This process plays a pivotal role in enhancing the systemic migration of administered cells toward sites of inflammation, along with the help of the pharmacological traits of MSCs with the biomimetic membrane and the overexpression of CD44 induced by HA. In this study, we successfully fabricated biomimetic nanoparticles named CPT@PLGA/MSC by coating the cell membranes of MSCs pre-treated with HA onto PLGA nanoparticles loaded with CPT. This work offered novel therapeutic prospects in the following process: After the administration of CPT@PLGA/MSCs, the overexpression of CD44 on their surfaces promoted the adhesion and rolling along the vessel wall by binding to selectins, facilitating the migration of CPT@PLGA/MSCs from distant areas to the vicinity of the inflammation (Figure 1) [38]. After this initial adhesion, CPT@PLGA/MSCs navigated through the extracellular matrix under the guidance of chemotactic signals released at the injury site and then migrated through the endothelial layer into the inflammation site by means of enhanced permeability and retention effect [54], where CPT@PLGA/MSCs could be further up taken by inflammatory cells, and then CPT was released into the cytoplasm, thereby exerting their therapeutic effects.

HA was introduced into the lower chambers of Transwells to evaluate its enhancing effect on the extracellular migration capability of MSCs. Stimulation resulted in a significant 2.86-fold increase in the extracellular migration of MSCs across the polycarbonate filter. MSCs displayed active migration in the presence of HA but reduced migration in the absence of HA. This finding is consistent with previous results [21,30] and highlights the role of HA in promoting MSC migration. Flow cytometry results revealed that, after stimulation with 30 μg/mL HA, the expression of CD44 on MSC membranes increased from 27.3% to 95.1%. Although the CD44-positive expression rates in the control group and the literature differed [28], our results confirmed the findings of previous research [30]. The characterization of CPT@PLGA/MSCs on the basis of morphology, particle size, zeta potential, encapsulation efficiency, and stability revealed that CPT@PLGA/MSCs had a uniform spherical morphology and a slightly increased particle size after membrane coating. The zeta potential of CPT@PLGA/MSC was in accordance with the requirements for intravenous administration. TEM imaging showed that the MSC membranes coated CPT@PLGA/MSC exclusively. These observations were supported by SDS–PAGE and Western blotting analyses. SDS–PAGE was performed to assess the presence of cell membrane coating on nanoparticles by examining the protein composition of isolated cell membranes and membrane-coated nanoparticles. Western blot analysis confirmed that specific protein markers were expressed on the stem cell membranes. After a week of storage at 4 °C, no significant changes were observed in the particle size, zeta potential, and PDI of CPT@PLGA/MSC, indicating that PLGA/MSC exhibited a good encapsulation effect on CPT, preventing leakage during storage. Furthermore, CPT@PLGA/MSC exhibited high EE and LC of 88.16% ± 1.77 and 4.76% ± 0.11, respectively. These results indicated the suitable preparation and satisfactory stability of the nanoparticles. The release profiles of CPT were indistinguishable between CPT@PLGA/MSC and CPT@PLGA, which suggested that the membrane coating could not affect growth drug releases from CPT@PLGA/MSCs [55]. The maximum cumulative release rates of CPT@PLGA/MSCs and CPT@PLGA were both below 50%, indicating that PLGA/MSCs exhibited effective encapsulation of the lipophilic CPT, minimizing drug leakage to a significant extent. Furthermore, a similar trend of the decrease of CPT in media after a certain period of time was observed in the free CPT group, CPT@PLGA group, and CPT@PLGA/MSC group. The underlying reason for this phenomenon may be attributed to the instability of CPT in the media. 

An oxidative stress model of LO2 cells was established using H_2_O_2_ to detect the biological activity of CPT [56]. The presence of antioxidant enzymes, such as CAT, indicated the progression of cellular oxidative stress and lipid peroxidation [57]. GSH, which is recognized as a vital antioxidant and detoxifying agent, serves as a key indicator in the assessment of cellular oxidative stress damage [58]. The exacerbation of cellular oxidative stress often coincides with related inflammatory responses [59]. In this paper, 20 μg/mL of CPT@PLGA and CPT@PLGA/MSC could alleviate oxidative stress and proinflammatory cytokine levels, affirming the applicability of CPT in the effective treatment of ALI. Further, a CCl_4_-induced mouse model was selected to further evaluate the in vivo liver targeting efficiency and effectiveness of CPT@PLGA/MSCs for ALI treatment [60]. Oxidative stress and inflammation are the main promoting factors of pathogenesis in CCl_4_-induced ALI. Numerous free radicals directly or indirectly inflict hepatic tissue damage by covalently binding to macromolecules and initiating lipid peroxidation. The released damage-associated molecular pattern molecules from these destroyed hepatocytes activate and recruit Kupffer cells (KCs) or macrophages, which produce excessive amounts of proinflammatory cytokines, leading to a second wave of amplified inflammation and resulting in severe hepatic damage or liver failure. Therefore, the inhibition of oxidative stress and inflammation could act as an effective therapeutic strategy for attenuating ALI [61]. The ex vivo imaging results revealed a significant enhancement in liver targeting after the introduction of MSC membranes. Due to pretreatment with HA, which led to the upregulation of the CD44 protein on MSC membranes, the liver-targeting capability of the Cy5.5@PLGA/MSC group was significantly enhanced compared to the Cy5.5@PLGA/MSC group (non-HA pretreated control). The frozen sections showed that PLGA primarily accumulated around KCs and was subsequently cleared. After the MSC-membrane coating, the nanoparticles evaded the capture of KCs and accumulated in hepatic stellate cells (HSCs). This escape was facilitated by the biomimicry characteristics of PLGA/MSC. The migration of PLGA/MSC to HSCs may help suppress their activation, which is closely linked to liver fibrosis [62], and potentially restore normal liver structure.

Subsequently, the in vivo therapeutic role of CPT@PLGA/MSCs in ALI was assessed by measuring various biochemical indicators in serum and liver tissue homogenates. Oxidative stress is recognized as a key contributor to a range of liver diseases [63,64]. CCl_4_ was commonly employed to study both acute and chronic liver injury [65]. This type of liver damage is marked by distinctive centrilobular necrosis, a significant surge in serum transaminases, increased lipid peroxidation, and compromised antioxidant defense mechanisms. The presence of excessive free radicals accelerates the degradation of cellular membranes, resulting in the loss of cell integrity and the release of ALT and AST into the bloodstream [65]. AKP, which is closely linked to the cell membranes of diverse tissues, particularly liver cells, provides direct insights into liver tissue damage [66]. The obtained serum biochemical indicators revealed that the CPT@PLGA/MSC group exhibited considerable reductions in serum AST, ALT, and AKP levels. GSH, SOD, and CAT effectively indicated the extent of tissue or cellular oxidative stress damage. MDA, a pivotal by-product of oxidative reactions, also served as an effective marker of oxidative stress [59]. In addition, a multitude of inflammatory cytokines were released after hepatocyte injury, including the proinflammatory cytokines IL-1β, IL-6, and TNF-α, triggering upregulated inflammatory responses [67]. CPT@PLGA and CPT@PLGA/MSCs were effective in reducing oxidative stress and proinflammatory cytokine levels in mice livers, with CPT@PLGA/MSCs providing greater therapeutic benefits. Moreover, H&E staining revealed a protective effect on liver cell morphological structure. In the present study, CCl_4_ induced severe hyperemia and congestion inside the portal area, along with leukocytic infiltration, hepatic degeneration, and bridge fibrosis. Although treatment with CPT@PLGA partially restored liver lobule structure, inflammation remained evident in the portal vein area. Conversely, the morphology of the liver of mice treated with CPT@PLGA/MSCs closely resembled that of a healthy liver, highlighting the therapeutic superiority of this formulation. In addition, preliminary in vivo safety experiments were conducted on CPT and the carriers. The histological analysis of major organs and serum biochemical indicators after administration showed no evidence of tissue damage or abnormalities. This suggested a favorable safety profile for both CPT and the carriers.

## 5. Conclusions

A novel nanoscale formulation featuring MSC membrane coatings was designed and developed for the targeted delivery of the anti-inflammatory drug CPT. The biomimetic nanoparticles, CPT@PLGA/MSCs, inherited the unique properties and intricate surface chemistry of natural cell membranes, thus exhibiting a specific homing effect that enables effective drug delivery to damaged areas within the body and thereby enhancing drug accumulation at the target site. CPT demonstrated excellent sustained-release capabilities and the full potential of its therapeutic effects after successive encapsulation by PLGA and MSC membranes. CPT@PLGA/MSCs effectively targeted the liver after intravenous injection. Histological and liver function tests revealed the ability of CPT@PLGA/MSC to alleviate CCl_4_-induced ALI in a mouse model. The findings of this work underscore the tremendous potential of CPT@PLGA/MSC as a biomimetic nanoparticle platform for the treatment of ALI and other inflammation-related diseases. 

## Figures and Tables

**Table 1:** Physicochemical properties of the prepared nanoparticles.

Sample	Particle Size (nm)	PDI	Zeta Potential (mV)
CPT@PLGA	183.9 ± 4.5	0.225 ± 0.048	−21.7± 0.7
MSC membrane vesicles	352.4 ± 18.5	0.695 ± 0.047	−10.5 ± 0.9
CPT@PLGA/MSC	204.3 ± 4.5	0.270 ± 0.028	−9.6 ± 0.9

## Data Availability

Data are contained within the article.

## Supplementary Materials

The following supporting information can be downloaded at: https://www.mdpi.com/article/10.3390/pharmaceutics15122764/s1, Figure S1: Study on HPLC methodology of CPT (A) The standard solution for CPT; (B) The emulsion-breaking solution of PLGA/MSC; (C) The mixed solution of CPT and PLGA/MSC; (D) The standard Curve of CPT. Both CPT and PLGA/MSC are dissolved in acetonitrile solution. Table S1: Precision Measurement Results. Table S2: Recovery Determination Results.
