# Supplementary material for: Targeted Therapy of Acute Liver Injury via Cryptotanshinone-Loaded Biomimetic Nanoparticles Derived from Mesenchymal Stromal Cells Driven by Homing
Source: Pharmaceutics. 2023 Dec 12;15(12):2764. doi: 10.3390/pharmaceutics15122764 (PMC10747007; doi:10.3390/pharmaceutics15122764)
Supplement: Supplementary file 1 [file pharmaceutics-15-02764-s001.zip › pharmaceutics-2696806-supplementary.pdf]

Supplemental Figure

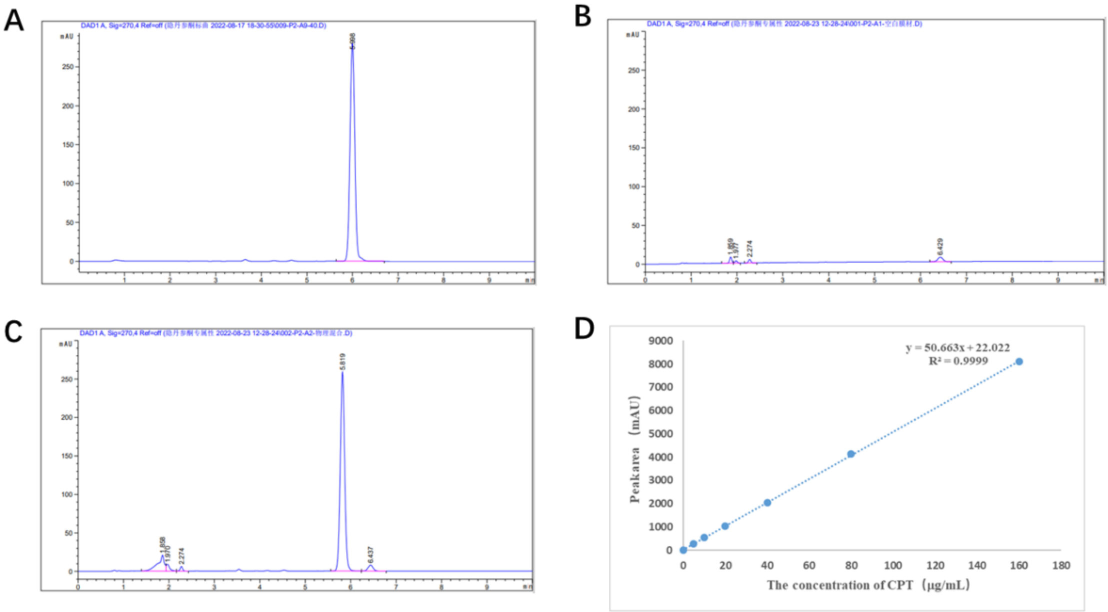

Supplemental Figure S1. Study on HPLC methodology of CPT (A) The standard solution for CPT;（B）The emulsion-breaking solution of PLGA/MSC;（C）The mixed solution of CPT and PLGA/MSC;（D）The standard Curve of CPT. Both CPT and PLGA/MSC are dissolved in acetonitrile solution.

Table S1. Precision Measurement Results.

measured concentration (μg/mL)	theoretical concentration (μg/mL)	Mean ± SD	RSD%
39.81	40.00	39.87 ± 0.43	0.10
39.87			
39.94			
39.86			
39.85			

Table S2. Recovery Determination Results.

theoretical concentration (μg/mL)	measured concentration (μg/mL)	Recovery(%) ± SD	RSD (%)
8.33	8.40 ± 0.103	100.80	1.23
33.33	32.96 ±0.184	98.90	0.56
66.67	67.14 ± 0.220	100.70	0.33
